# Modelling Analysis of COVID-19 Transmission and the State of Emergency in Japan

**DOI:** 10.3390/ijerph18136858

**Published:** 2021-06-26

**Authors:** Zhongxiang Chen, Zhiquan Shu, Xiuxiang Huang, Ke Peng, Jiaji Pan

**Affiliations:** 1 College of Engineering and Design, Hunan Normal University, Changsha 410081, China; chenzx@hunnu.edu.cn (Z.C.); hxxpxj01@163.com (X.H.); pengke@hunnu.edu.cn (K.P.); 2 School of Engineering and Technology, University of Washington, Tacoma, WA 98402, USA; zqshu@uw.edu; 3 State Key Laboratory of Developmental Biology of Freshwater Fish, Hunan Normal University, Changsha 410081, China

**Keywords:** COVID-19, mathematical modelling, state of emergency, containment policies

## Abstract

To assess the effectiveness of the containment strategies proposed in Japan, an SEIAQR (susceptible-exposed-infected-asymptomatic-quarantined-recovered) model was established to simulate the transmission of COVID-19. We divided the spread of COVID-19 in Japan into different stages based on policies. The effective reproduction number $R_{e}$ and the transmission parameters were determined to evaluate the measures conducted by the Japanese Government during these periods. On 7 April 2020, the Japanese authority declared a state of emergency to control the rapid development of the pandemic. Based on the simulation results, the spread of COVID-19 in Japan can be inhibited by containment actions during the state of emergency. The effective reproduction number $R_{e}$ reduced from 1.99 (before the state of emergency) to 0.92 (after the state of emergency). The transmission parameters were fitted and characterized with quantifiable variables including the ratio of untracked cases, the PCR test index and the proportion of COCOA app users (official contact confirming application). The impact of these variables on the control of COVID-19 was investigated in the modelling analysis. On 8 January 2021, the Japanese Government declared another state of emergency. The simulated results demonstrated that the spread could be controlled in May by keeping the same strategies. A higher intensity of PCR testing was suggested, and a larger proportion of COCOA app users should reduce the final number of infections and the time needed to control the spread of COVID-19.

## 1. Introduction

The coronavirus disease (COVID-19) has continued to spread since the first recorded case at the end of 2019 [1,2]. The global cumulative number of reported confirmed cases has risen to over 168 million with 3.6 million deaths [3].

A number of containment strategies have been enforced to control the pandemic by many governments including restrictions on travel and public gatherings, school delays or closings, social distancing, and even lockdown measures [4,5,6,7,8]. Some extreme actions can have a significant impact on the socioeconomic systems [9], which may be continued by some authorities [10]. Thus, it is of importance to optimize the containment policies to stop the spread.

The COVID-19 infection in Japan started in the middle of January 2020 [11]. The Japanese government put forward basic control policies in February and March to curb the early transmission [12]. As the number of confirmed cases rapidly grew, and a large portion of infections could not be tracked, a state of emergency was declared by the central government on 7 April 2020 [12]. After the termination of the first state of emergency in Japan, the spread of COVID-19 was not well controlled. The number of newly confirmed cases quickly rose at the end of 2020. The number of cumulative confirmed cases in Japan hit 401,355 as of 6 February 2021 [13]. Another state of emergency was declared in early 2021 [14].

To assess the containment policies and evaluate the effectiveness of the state of emergency, appropriate modelling studies could offer useful instruction and guidance. A study adopted the susceptible–infected–removed (SIR) transmission model to analyze the effectiveness of the early strategies proposed by the Japan Government [6]. It was concluded that it was necessary to minimize the length of time people stayed in crowded places. However, the study did not highlight testing or isolation measures. In addition, the spread of COVID-19 was affected by the time-varying policies. Thus, the parameters associated with the model should be dynamic, and should be estimated accurately. Another study utilized a susceptible–exposed–infected–recovered (SEIR) model to forecast the peak time of transmission in Japan. However, the rate of transmission from the exposed population was not taken into consideration [15]. Moreover, the infectivity of the large portion of asymptomatic patients should not be neglected.

The SEIR-like models were also applied in some other studies for the analysis of the transmission of COVID-19 and of the control policies [16,17,18,19]. Jiao considered that patients were contagious during the incubation period [17], which was underestimated in many models. It was demonstrated that isolation should be strictly carried out to curb the pandemic. A modified SEIRV model examined the effect of environmental conditions, in which V represented the external concentration of the virus. The conclusion was drawn that long-term strategies were necessary since COVID-19 would, otherwise, remain endemic according to the simulation.

In this study, we established a susceptible–exposed–infected–asymptomatic–qurantine–recovered/removed (SEIAQR) model. Asymptomatic infection [20,21,22] was especially emphasized in the model. The policies of isolation measures and the Polymerase Chain Reaction (PCR) test [23] were quantitatively characterized and analyzed. The spreading period of COVID-19 was partitioned into several stages according to the containment policy announcements, including the declaration of the state of emergency. The parameters in the model associated with the dynamics of COVID-19 transmission were determined by optimizing and fitting the simulated results with the actual number of infected cases reported.

Based on the simulation results, the declaration of the state of emergency was effective, as shown by the reductions in the coefficients of transmission rates and the basic reproduction number. Then, we used the established model to analyze the second declaration of a state of emergency. Finally, the strategies, with different intensities of isolation, PCR test index and the percentage of users of tracking applications, were optimized to offer some instructions to control the COVID-19 pandemic.

## 2. Methods

### 2.1. Model Description

**Figure 1:**
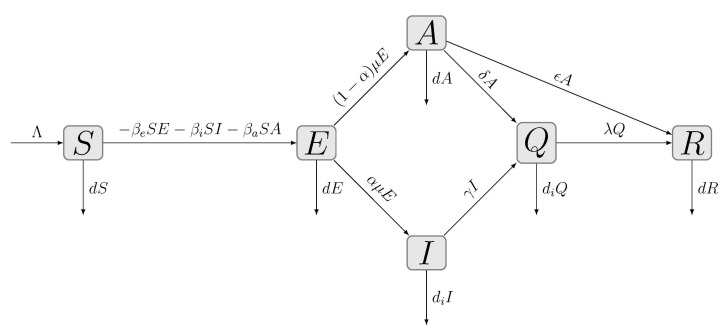
The graphical illustration of the SEIAQR model.

The compartmental model established for this study is illustrated in Figure 1. The compartments included susceptible (denoted by *S*), exposed (denoted by *E*), infected (denoted by *I*), asymptomatic (denoted by *A*), quarantined (denoted by *Q*) and recovered/removed (denoted by *R*) populations. The dynamics of the spread of COVID-19 and the interactions between compartments were characterized by the system of Equation (1) in this model. Specifically, asymptomatic infection and infectivity during the incubation period (exposed compartment) were taken into consideration, which improved the accuracy for simulating COVID-19 transmission.

**Figure 2:**
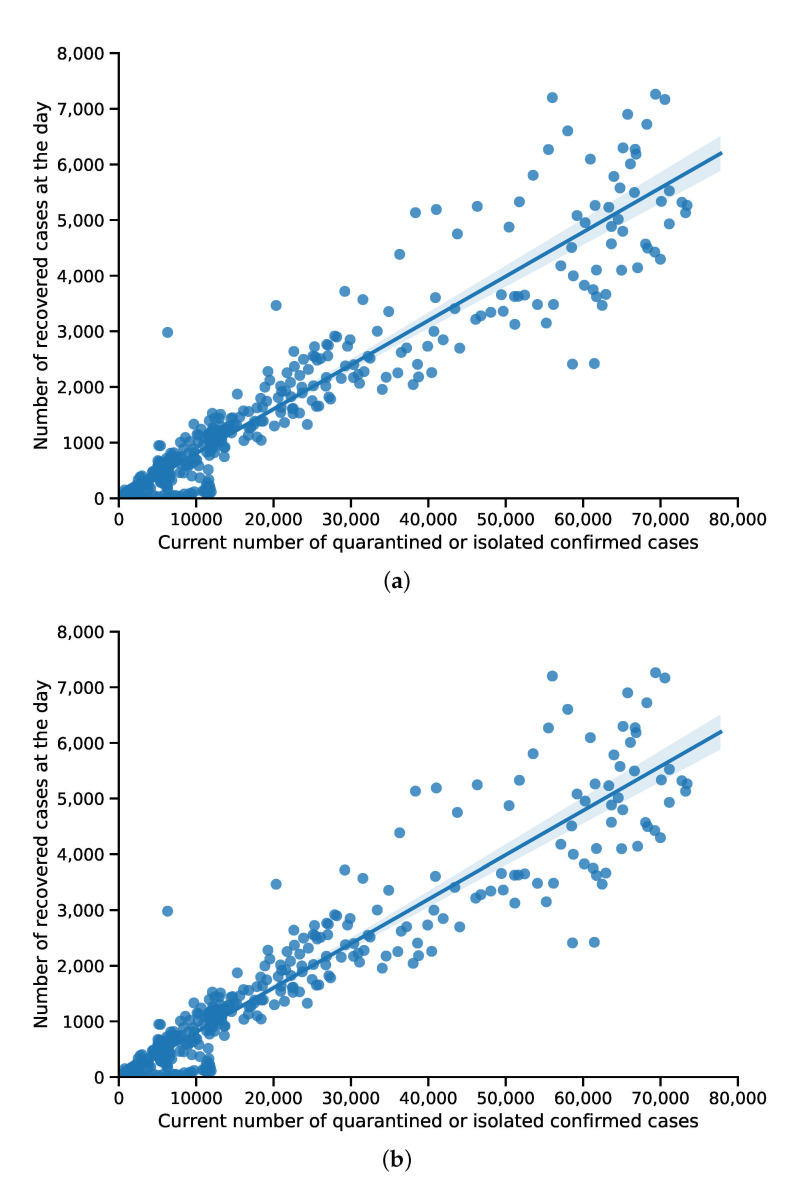
Data fitting for the recovery rate and death rate of COVID-19 in Japan. (**a**) Data fitting for the recovery rate. (**b**) Data fitting for the death rate.

The structure and dynamics of COVID-19 transmission are shown in Figure 1, and the discrete dynamic system is

(1) $$\left\{\begin{array}{c} S\left(t+1\right)=S\left(t\right)+\Lambda -\beta_{e}S\left(t\right)E\left(t\right)-\beta_{i}S\left(t\right)I\left(t\right)-\beta_{a}S\left(t\right)A\left(t\right)-dS\left(t\right)\\ E\left(t+1\right)=E\left(t\right)+\beta_{e}S\left(t\right)E\left(t\right)+\beta_{i}S\left(t\right)I\left(t\right)+\beta_{a}S\left(t\right)A\left(t\right)-\mu E\left(t\right)-dE\left(t\right)\\ I\left(t+1\right)=I\left(t\right)+\alpha \mu E\left(t\right)-\gamma I\left(t\right)-d_{i}I\left(t\right)\\ A(t+1)=A(t)+(1-\alpha )\mu E(t)-\delta A(t)-\varepsilon A(t)-dA(t)\\ Q\left(t+1\right)=Q\left(t\right)+\delta A\left(t\right)+\gamma I\left(t\right)-\lambda Q\left(t\right)-d_{i}Q\left(t\right)\\ R(t+1)=R(t)+\varepsilon A(t)+\lambda Q(t)-R(t), \end{array}\right.$$

where *S*, *E*, *I*, *A*, *Q* and *R* represent the proportion of individuals that are susceptible, exposed, asymptomatic, documented and recovered/removed at time *t* (discrete time). The transmission rates from the compartments *E*, *I* and *A* are denoted by $\beta_{e}$, $\beta_{i}$ and $\beta_{a}$ (per day), respectively. Λ (the natural birth rate) is about $2.0\times 10^{-5}$[24]. *d* and $d_{i}$ denote the natural death rate and the COVID-19 death rate in Japan, respectively. *d* was set to be $3.0\times 10^{-5}$[24] and $d_{i}$ was determined as $1.4\times 10^{-3}$ by fitting the ratio of deaths to the number of confirmed cases (Figure 2a). The incubation period $\frac{1}{\mu }$ was set to be 5 days [15] (μ is the rate of transmission from the exposed population to the infected individuals with symptoms). α and 1−α represent the proportion of infected patients with symptoms or asymptomatic patients. γ and δ denote the confirmation rate of the symptomatic infections and asymptomatic infections, respectively. ε (per day) is the recovery rate of asymptomatic infections and λ (per day) is the recovery rate of confirmed infections. The average recovery times $\frac{1}{\varepsilon }$ ( ε is the recovery rate for symptomatic infected patients) and $\frac{1}{\lambda }$ ( λ is the recovery rate for asymptomatic infected patients) were determined to be 11.5 days by data fitting (Figure 2b).

The confirmed asymptomatic or symptomatic cases were presumed to be isolated and thus could not spread COVID-19. The reproduction number $R_{e}$, the expected value of secondary infections produced by one typical infected case, was characterized as the following [25]:

(2) $$R_{e}=\beta_{e}\frac{\Lambda }{d(\mu +d)}+\beta_{i}\frac{\alpha \mu \Lambda }{d\left(\gamma +d_{i}\right)\left(\mu +d\right)}+\beta_{a}\frac{(1-\alpha )\mu \Lambda }{d(\delta +\epsilon +d)(\mu +d)}.$$

### 2.2. Determination of the Transmission Parameters

The parameters $\beta_{e}$, $\beta_{i}$, $\beta_{a}$, α, γ, δ needed to be determined based on the real confirmed cases. Usually, the least-squares method is adopted to obtain the best fitting values based on the number of daily confirmed cases. However, the data on the daily reported confirmed cases for COVID-19 fluctuated greatly as can be seen in Figure 1. On the contrary, the change of cumulative confirmed cases was smooth. Therefore, the cumulative confirmed cases were utilized to calculate these parameters.

Set $Y_{r}\left(t\right)$ as the actual cumulative confirmed cases at time *t*, where $t\in Z^{+}$ and

(3) $$Y_{r}\left(t\right)=\sum_{\tau =0}^{t}D\left(\tau \right),$$

where D(τ) is the daily confirmed cases on $\tau \in Z^{+}$. Set $Y_{h}\left(t\right)$ as the numerical cumulative confirmed cases at time *t* by solving the SEIAQR model (1), thus

(4) $$Y_{r}\left(t\right)=Y_{h}\left(t\right)+\xi \left(t\right),$$

where ξ(t) is the error at time *t* and $Y_{h}\left(t\right)$ is expressed as

(5) $$Y_{h}\left(t\right)=\sum_{\tau =0}^{t}\left(\gamma I(\tau )+\delta A(\tau )\right).$$

Then, the problem of solving the parameters $\beta_{e}$, $\beta_{i}$, $\beta_{a}$, α, γ, δ was transformed into a nonlinear square optimization problem as

(6) $$\min\sum_{\tau =0}^{t}\xi^{2}\left(t\right)=\min\sum_{\tau =0}^{t}{\left(Y_{r}\left(t\right)-Y_{h}\left(t\right)\right)}^{2},\;\;\mathrm{for}\;\;0<\beta_{e},\beta_{i},\beta_{a},\alpha ,\gamma ,\delta <1.$$

### 2.3. COVID-19 Data and Stages for Analysis

**Figure 3:**
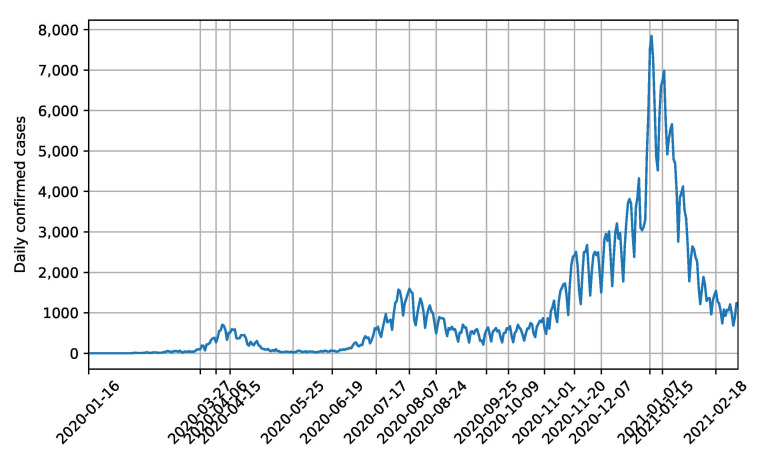
Segregation of the spread of COVID-19 in Japan for the modelling study.

In this modelling study, the data were collected from the official website of the Japanese Ministry of Health, Labour and Welfare, including a reported confirmed number of symptomatic or asymptomatic cases, PCR testing, and so forth [26]. The COVID-19 pandemic timeline in Japan was divided into 16 stages, primarily based on the policies proposed by the government (Table 1). The timeline with 16 stages is shown in Figure 3. The dates segregating the stages are marked on the horizontal axis. The transmission parameters were determined dynamically in each stage. In particular, the emphasis in this study was on the analysis of the stages that involved the state of emergency.

## 3. Results

### 3.1. Simulated Results and Determined Model Parameters

**Figure 4:**
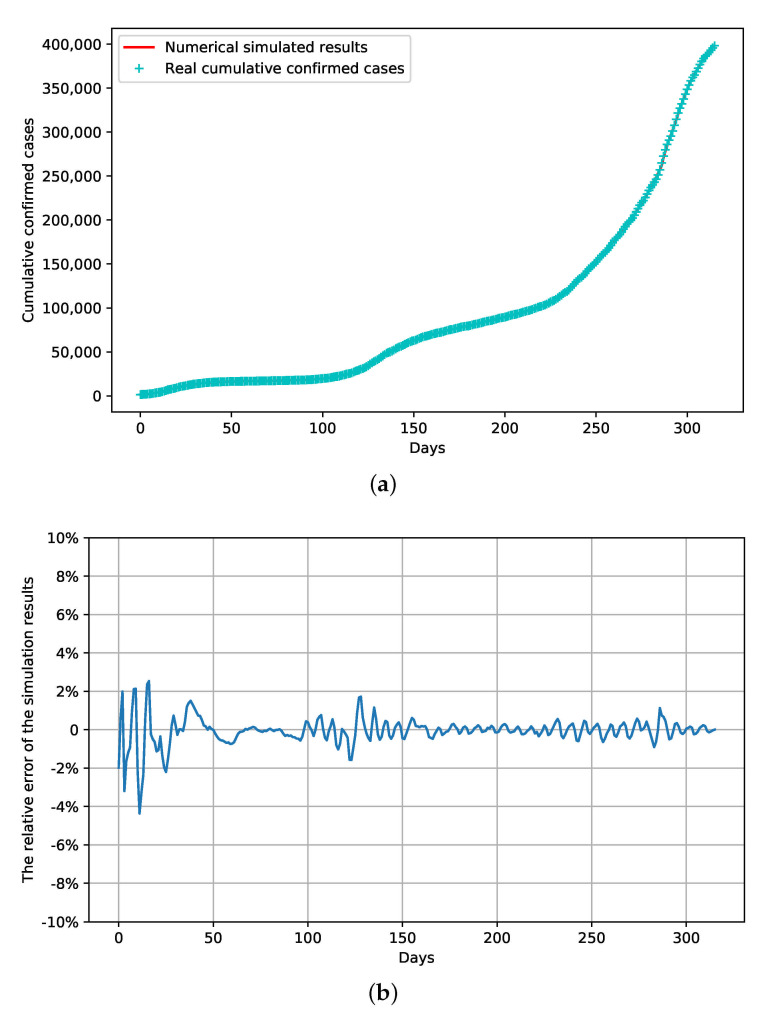
The simulation of confirmed cases over time by the model. (**a**) The simulated number of cases fitted well with the real accumulated confirmed cases. (**b**) The relative error between the simulated and real data.

The simulation of the spread of COVID-19 in Japan was performed using the established model. With the transmission parameters obtained (Table 2), the development of the number of confirmed cases over time fitted well with the reported data (Figure 4a). The relative error between the simulated and real number of confirmed cases did not exceed 5% in all the stages (Figure 4b). The effective reproduction numbers ($R_{e}$) during the stages were calculated with a 95% confidence interval (CI) as shown in Table 3.

Upon the outbreak of COVID-19 in Japan, the transmission parameters $\beta_{e}$, $\beta_{i}$ and $\beta_{a}$ were 0.0858, 0.2183 and 0.3217, respectively. In stage 2, when the basic policies were announced, the transmission parameters were $\beta_{e}=0.1635$, $\beta_{i}=0.4668$ and $\beta_{a}=0.5562$, respectively. The parameters were not lowered, illustrating that early containment measures did not control the disease spread. The confirmation rates increased in stage 2, suggesting a higher proportion of cases were tracked by testing. The effective reproduction number $R_{e}$ increased from 1.52 (95% CI 1.47–2.12) to 1.99 (95% CI 1.90–2.27). After declaring the first state of emergency (stages 3 and 4), the transmission parameters were lowered to $\beta_{e}=0.0849$, $\beta_{i}=0.1933$ and $\beta_{a}=0.2357$ (stage 3); $\beta_{e}=0.0534$, $\beta_{i}=0.1929$ and $\beta_{a}=0.1800$, respectively (stage 4). The reproduction number $R_{e}$ declined to 0.92 (95% CI 0.83–1.00) in stage 3 and 0.59 (95% CI 0.53–0.61) in stage 4. Although a previous study suggested that following the early basic policies proposed in stage 2 could control the transmission of COVID-19 [40], the rapid spread of COVID-19 was not contained until taking effective measures during the state of emergency.

After the first state of emergency was lifted, the spread of COVID-19 continued to develop with reproduction numbers higher than 1 except for in stages 8 and 9 (Table 3). In stage 8, the PCR testing intensity was reinforced [32]. This may have resulted in a more effective control of the spread of COVID-19, compared to the previous stages. In stage 6, an application used for tracking the cases was adopted. The gradual increase of users of the app should play a positive role in controlling the disease.

By the end of 2020, the second wave of infections occurred in Japan. Another state of emergency was declared by the Japanese government on 7 January 2021. After declaring the second state of emergency (stages 15 and 16), the transmission parameters decreased from $\beta_{e}=0.0633$, $\beta_{i}=0.2795$, $\beta_{a}=0.1932$ (stage 14) to $\beta_{e}=0.0489$, $\beta_{i}=0.1721$ and $\beta_{a}=0.1897$ (stage 15), respectively; $\beta_{e}=0.0444$, $\beta_{e}=0.1613$ and $\beta_{e}=0.1585$ (stage 16), respectively. The reproduction number Re reduced to 0.91 (95% CI 0.89–1.90) in stage 15 and 0.67 (95% CI 0.38–0.71) in stage 16. Compared to the first state of emergency in stage 4, the $R_{e}$ was slightly larger and the confirmation rate was smaller, which may possibly be due to the different intensities of the distancing strategies or variations in the virus’ infectivity [41]. The proportion of asymptomatic cases ranged from less than 10% to over 80% based on different reports [42]. In this model, the proportion of asymptomatic cases ranged from 67% to 90% in various stages (Table 2).

### 3.2. Fitting of the Transmission Parameters

**Figure 5:**
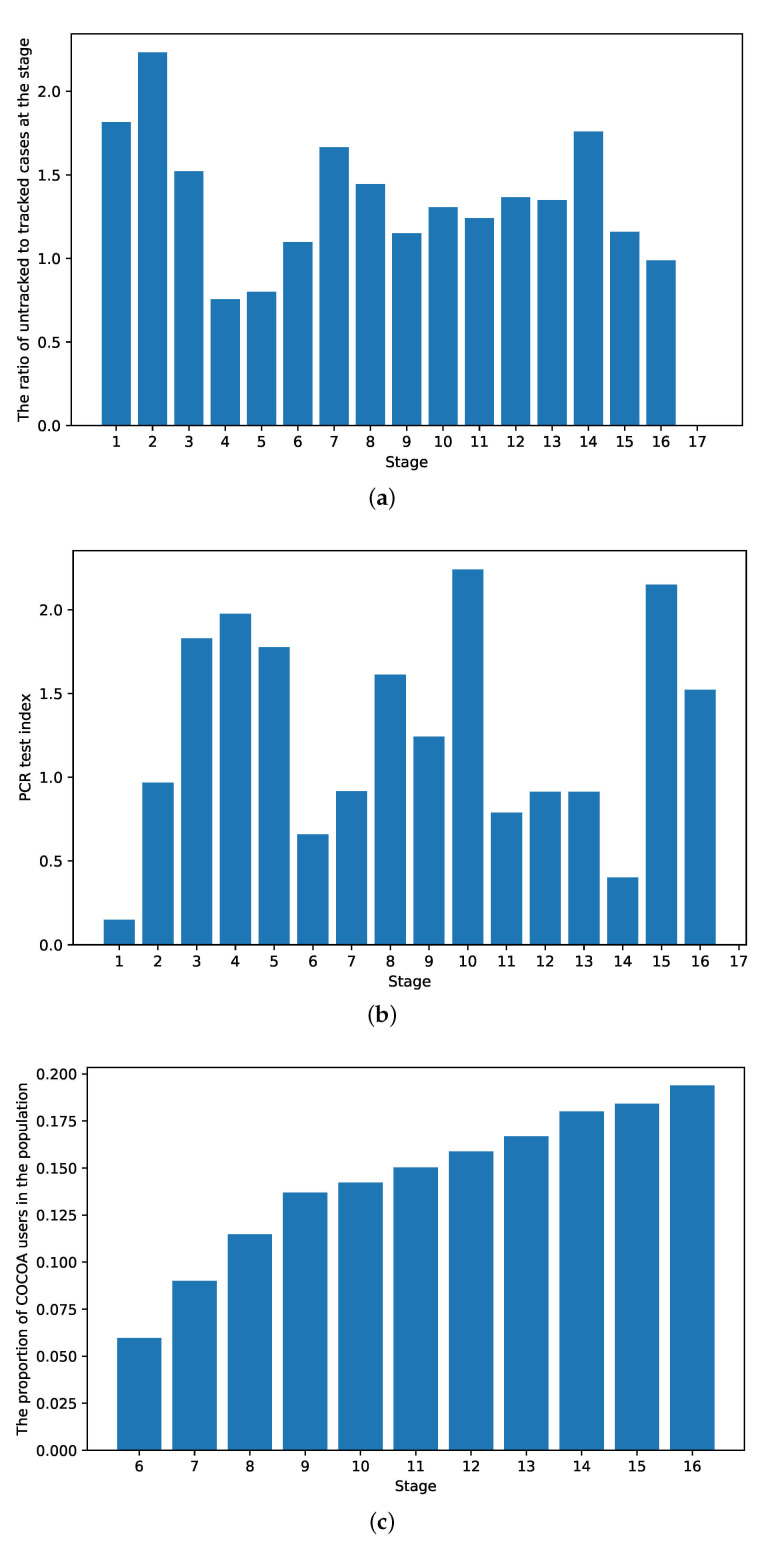
The variables for the fitting of the model parameters and characterization of the strategies. (**a**) The ratios of untracked to tracked cases in the stages. (**b**) PCR test indexes in the stages. (**c**) The proportions of COCOA app users in the stages.

To investigate the impact of various strategies on the control of the COVID-19 spread, the transmission rates $\beta_{e}$, $\beta_{i}$ and $\beta_{a}$ were fitted by the defined variables related to the containment policies. The variables used in the fitting included the ratio of untracked to tracked cases at stage *p*, the PCR test index χ and the proportion of COCOA (contact-confirming application) users in the population η. The official contact-confirming application was put forward in stage 6 and the percentage of users gradually increased. The values of the variables across the stages are shown in Figure 5.

By fitting the transmission parameters with the ratio of untracked to tracked cases *p*, the PCR test index χ, the proportion of COCOA users in the population η and the confirmation rate of symptomatic infected cases γ, the following equations were determined:

(7) $$\beta_{e}=e^{-2.79+0.72\gamma -0.92\gamma^{2}-0.57p+0.23p^{2}+3.72\chi -3.69\chi^{2}+0.96\chi^{3}-3.44\eta },$$

(8) $$\beta_{i}=e^{-0.63+0.37\gamma -0.91\gamma^{2}-1.78p+0.61p^{2}+3.25\chi -3.08\chi^{2}+0.75\chi^{3}-1.72\eta },$$

(9) $$\beta_{a}=e^{-1.70+2.44\gamma -2.71\gamma^{2}-0.96p+0.39p^{2}+3.24\chi -3.39\chi^{2}+0.93\chi^{3}-2.45\eta }.$$

The $R^{2}$ (goodness-of-fit measure) for the multivariable regressions were 0.926, 0.917, 0.970 and 0.939, respectively. The confirmation parameters δ and γ are associated with the testing. The infected cases with symptoms could be more easily identified but the asymptomatic patients could hardly be identified other than with PCR testing, which should result in difficulties for disease control. The confirmation rate δ characterized the proportion of determined asymptomatic cases by testing the estimated total asymptomatic cases. The confirmation rate δ was fitted for the 16 stages in the following:

(10) $$\delta =\frac{1}{1+e^{-1.06-1.87\gamma +2.65\gamma^{2}+3.65p-1.16p^{2}-2.07\chi +1.74\chi^{2}-0.43\chi^{3}+3.81\eta }}.$$

The $R^{2}$ for the multivariable regressions was 0.938. It was found that the confirmation rate could be increased by a higher proportion of COCOA app users. Furthermore, the effects of *p* and χ were investigated in the following section. The strategies used in stage 16 were optimized by this model.

Given the previous analysis, in the current stage with enhanced testing parameters but where the basic production number has not been reduced to less than 1, we may consider using a combination of increased isolation level and maintaining a moderate testing intensity.

### 3.3. Prediction of Cumulative Confirmed Cases and Effect of Combined Strategies

**Figure 6:**
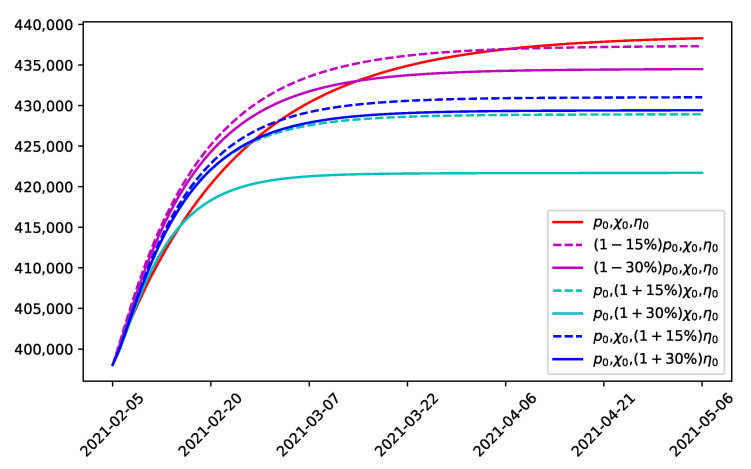
The prediction of the development of cumulative confirmed cases when changing the intensity of the strategies. Red line represents using the current strategy in the state of emergency; Pink lines represent using the modified strategies and lowering the ratio of untracked cases; cyan lines represent using the modified strategies with strengthened PCR tests; blue lines represent using the modified strategies alongside promoting the COCOA application.

In this section, the SEIAQR model was used to predict the spread of COVID-19 the current containment strategies in stage 16 are maintained or modified strategies are adopted. On the basis that the simulation of the previous stages fitted well with the trend of real reported confirmed cases (Figure 4), the impact of the ratio of untracked to tracked cases *p*, the PCR test index χ and the proportion of COCOA users in the population η on the development of predicted cumulative cases was analyzed by numerical simulation (Figure 6), which provided a sensitivity analysis of the parameters for the model and instructions to optimize the combination of control strategies.

By maintaining the same current containment strategy in stage 16 (the second state of emergency), the cumulative number of infections would reach a stable peak in early May. The predicted cumulative number of confirmed cases would be around 438,000 (red line in Figure 6). If the containment strategies were modified with the same ratio of untracked cases p=0.9, the proportion of COCOA users as η=0.19 and with an increase in the intensity of the PCR testing index χ=1.5 by 15% or 30% (dashed or solid cyan lines in Figure 6), the epidemic could be effectively controlled by mid-March or early March. The final number of cumulative infections would be around 428,900 and 421,700, respectively.

At the current stage, the proportion of COCOA users is 19.3%. If the proportion η was increased to 22.2% or 25.1% while keeping the same p=0.9 and χ=1.5 (dashed or solid blue lines), the epidemic would be effectively controlled in mid-April or late March. The final number of cumulative infections would be around 428,900 and 421,700, respectively. The dissemination of disease information could interact with the spread of the disease [43,44]. It was found that promoting the usage of the COCOA application regarding disease information could help to control the spread of COVID-19. If *p* was decreased by 15% or 30% (dashed or solid pink lines), the effect on controlling the disease spread was not as significant as strengthening the PCR testing or promoting the application. The number of cumulative infections at the peak would be around 428,900 and 421,700, respectively.

**Figure 7:**
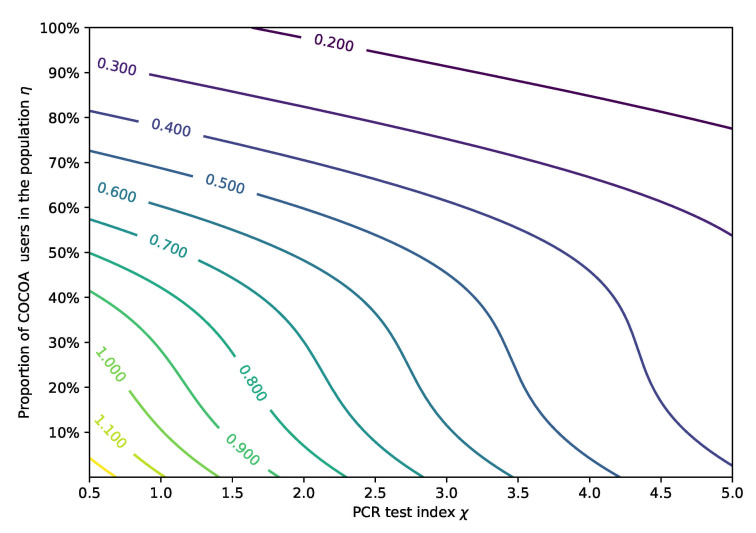
The contour of reproduction number $R_{e}$ with different η and χ.

The contour of the effective reproduction number $R_{e}$ was plotted as a function of the proportion of COCOA users in the population η and the PCR test index χ with the other parameters kept the same as in stage 16 (Figure 7). These contour lines demonstrated that Re decreased as the parameter values η and χ increased. In order to control the development of the epidemic ($R_{e}<1$), the parameters η and χ must be greater than 30% and 1.8, respectively. To achieve better control than the current state of emergency (stage 16), η must be increased by more than 0.65 and χ should be greater than 3.5. The combined control strategy in the following stages should be evaluated conveniently based on the contour.

### 3.4. Verification of the Model

**Figure 8:**
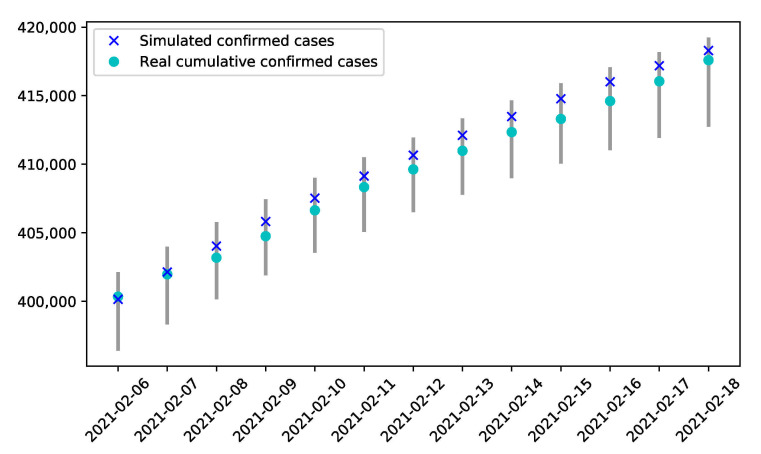
Verification of the model by comparing the predicted and real number of infections.

The data from 15 January 2020 to 5 February 2021 were used for the previous fitting and analysis. To validate the model, the number of confirmed cases from 6 February 2021 to 18 February 2021 was predicted by using the same configurations as in stage 16. As can be seen in Figure 8, under the assumption that the containment strategies should not change during the second state of emergency, the real data on the number of cumulative infections fell in the range of 95% CI of the cumulative confirmed cases predicted by the model. Thus, in addition to the fitting of the data (Figure 3), this model was further validated. The strategies during the second state of emergency could be optimized as guided by the model.

## 4. Discussion

An SEIAQR model was established to analyze the spread of COVID-19 in Japan and to evaluate the policies to control the pandemic. Generally, SIR or SEIR-like models [6,17,20] are composed of three or four compartments. In contrast, this study adopted six compartments, emphasizing the impact of asymptomatic infection. Infectivity during the incubation period, the rates of natural deaths and deaths due to COVID-19 were incorporated into the system to improve the reliability and authenticity of the model, although this resulted in more difficulties for the determination of the parameters. The proportion of asymptomatic patients was reported as ranging from less than 10% to over 80% based on different reports [42]. The estimation of the asymptomatic percentage ranges from 67% to 90% in this modelling study, which agrees with the previous experimental studies.

The transmission of COVID-19 was divided into 16 stages primarily determined by the policies put forward by the Japanese Government. The declaration of the state of emergency (stages 3, 4, 15, 16) could inhibit the rapid development of the disease spread since the effective reproduction numbers $R_{e}$ were less than 1. The strategies in the state of emergency were effective, which could be shown by the decrease of the transmission parameters. After the lifting of the state of emergency, the reproduction numbers $R_{e}$ became larger than 1. However, in stages 8 and 9, COVID-19 was better controlled compared to the other stages where there was no state of emergency declared, which was probably due to a higher PCR test intensity.

Many variables should affect the spread of COVID-19 but are rarely measured. We selected quantifiable indexes including the ratio of the untracked to tracked cases, the PCR test index and the proportion of COCOA app users. The transmission rates and confirmation rates of asymptomatic patients were fitted using these quantifiable variables. Then, for the second declaration of the state of emergency, the number of cumulative cases was predicted. It was found that increasing the PCR test index by 30% could decrease the number of overall infected cases by around $3\times 10^{4}$. The state of emergency could also be lifted earlier. Although social distancing measures are critical [45], it was found that increasing the PCR test index should have a more significant effect, followed by the impact of promoting the COCOA app. As suggested in another study, a higher intensity of testing should be implemented during the early spread period [46].

It was inferred that herd immunity in Japan could be achieved without sacrificing the current healthcare system if the basic reproduction number is less than 2 [47]. Our simulation revealed that, under the state of emergency, the reproduction number could be reduced to less than 1. After the lifting of the state of emergency, the reproduction number should be controlled at less than 1 by keeping a relatively high PCR test index and increasing the proportion of COCOA app users.

This study could be improved in the future. First, it was assumed that permanent immunity was achieved after recovery and the impact of the vaccine was also not taken into consideration. The compartment of the immune population could be included in the future. In addition, the stages were segregated based on policies. The stages could be divided with multiple approaches, such as using additionally defined indicators [48] to better evaluate the status of the spread. The transmission parameters could be estimated more accurately since the impact of policies may have some delay. Lastly, we excluded the impact of policies announced by local governments [49], which may result in some deviations in the fitting of parameters.

## Figures and Tables

**Table 1:** The stages of the spread of COVID-19 in Japan.

Stage	Period	Note
1	2020.01.06–03.27	The first case appeared in Japan [11].
2	2020.03.28–04.06	A series of basic policies were announced [27].
3	2020.04.07–04.15	Declared the state of emergency in several prefectures [28].
4	2020.04.16–05.25	Declared the first nationwide state of emergency [29].
5	2020.05.26–06.18	Lifted the state of emergency [12].
6	2020.06.19–07.16	Promoted cellphone app for contact information [30].
7	2020.07.17–08.07	Started saliva PCR testing to detect asymptomatic infection [31].
8	2020.08.08–08.24	Strengthened the testing intensity [32].
9	2020.08.25–09.25	Announced employment subsidy [33].
10	2020.09.26–10.08	Extended applications for subsidies for business suspension [34].
11	2020.10.09–11.01	Ensured the vacation and welfare of the patients [35].
12	2020.11.02–11.20	Abandoned two-week quarantine policy upon entry [36].
13	2020.11.21–12.07	Signed an agreement on provision of information sharing of cluster countermeasures for COVID-19 [37].
14	2020.12.08–2021.01.07	Announced to send additional medical staffs [38].
15	2021.01.08–01.15	Declared another state of emergency in several prefectures [39].
16	2021.01.16–02.18	Declared the second nationwide state of emergency [40].

**Table 2:** Parameters in different stages fitted by the model.

Stage	$\beta_{e}$ (per day)	$\beta_{i}$ (per day)	$\beta_{a}$ (per day)	α	γ	δ
1	0.0858	0.2183	0.3217	0.1	0.5244	0.2057
2	0.1635	0.4668	0.5562	0.1712	0.6374	0.3701
3	0.0849	0.1933	0.2357	0.2074	0.4771	0.3829
4	0.0534	0.1929	0.1800	0.2274	0.4603	0.5485
5	0.0971	0.3404	0.3103	0.1232	0.3188	0.3695
6	0.1213	0.4235	0.3864	0.1295	0.3238	0.3304
7	0.1015	0.3625	0.3175	0.1251	0.3839	0.2930
8	0.0335	0.1394	0.1371	0.1002	0.6536	0.1275
9	0.0478	0.1317	0.1165	0.1019	0.9107	0.1020
10	0.0491	0.1305	0.1976	0.1128	0.1374	0.1877
11	0.0742	0.2187	0.2113	0.1121	0.7453	0.1655
12	0.0867	0.2727	0.2929	0.2215	0.4233	0.1700
13	0.0724	0.2574	0.1623	0.1006	0.9378	0.1273
14	0.0633	0.2795	0.1932	0.1042	0.0588	0.1625
15	0.0489	0.1721	0.1897	0.1001	0.5687	0.1969
16	0.0444	0.1613	0.1585	0.1000	0.6152	0.2652

**Table 3:** Effective reproduction number $R_{e}$ determined by the model.

Stage	$R_{e}$	95% CI	Stage	$R_{e}$	95% CI
1	1.52	(1.47, 2.12)	9	0.86	(0.83, 0.88)
2	1.99	(1.90, 2.27)	10	1.03	(0.96, 1.12)
3	0.92	(0.83, 1.00)	11	1.2	(1.16, 1.26)
4	0.59	(0.53, 0.61)	12	1.52	(1.46, 1.63)
5	1.23	(1.19, 1.27)	13	1.12	(1.01, 1.15)
6	1.61	(1.56, 1.67)	14	1.44	(1.31, 1.92)
7	1.39	(1.37, 1.44)	15	0.91	(0.89, 1.90)
8	0.81	(0.79, 0.89)	16	0.67	(0.38, 0.71)

## Data Availability

The data, such as the number of positive cases, the number of people performing a PCR test, the number of people requiring inpatient treatment, the number of persons discharged or recovered, the number of the dead and the number of PCR tests performed can be accessed online at https://www.mhlw.go.jp/stf/covid-19/open-data.html. The data on new untraceable cases are available online at https://stopcovid19.metro.tokyo.lg.jp/en/cards/untracked-rate. The data on COCOA users are available online at https://www.mhlw.go.jp/stf/seisakunitsuite/bunya/cocoa_00138.html.
